# On the Use and Abuse of Purgatives

**Published:** 1819-12

**Authors:** 


THE LONDON

### Medical and Physical Journal.

6 OF VOL. XLII.] DECEMBER, 1819. [NO. 250.

"For many fortunate discoveries in medicine, and for the detection of numerous "errors, the world is indebted to the rapid circulation of Monthly Journals; "and there never existed any work to which the Faculty in EUROPE and "AMERICA were under deeper obligations than to the Medical and Physical "Journal of London, now forming a long, but an invaluable, series." RUSH.

### Original Communications.

FOR THE LONDON MEDICAL AND PHYSICAL JOURNAL.

### *On the Use and Abuse of Purgatives.* By Dr. KINGLAKE.

THE use of purgative remedies is most extensive in alleviating and curing diseases of various descriptions. There is, perhaps, no function in the animal economy of so much importance to life and health as the regular and uninterrupted office of the intestinal canal: any considerable deviation from the healthy action of the bowels must necessarily be productive of injurious effects. The first passages, as they are phrased, comprising the stomach and the whole alimentary tube, may be said to constitute the grand outwork of the animal frame: on the integrity, therefore, of this radical part of the system, will mainly depend the more or less perfect state of the general health. The diseases to which the stomach and bowels are liable, are various in nature, in form, and in degree; and all of them are of sufficient importance to demand early and efficient attention. Diseases of the inflammatory kind occurring in the first passages require immediate and adequate relief, to obviate the direct danger of life with which they are inseparably attended. Inflammatory affections of these parts must be speedily overcome, or there will be imminent risk of their running into the gangrenous state, with an insidious rapidity that would preclude the possibility of relief.

Diseases of this species may be regarded of the acute kind, and are liable to occur in any temperament, but more particularly in the sanguineous, the robust, and intemperate. The urgent distress attending these cases allows of no delay in resorting to some mode of remedy; it will therefore, in general, be as promptly rendered as required, and the desired relief will most likely be obtained. On these occasions bleeding is the principal remedy: it should be freely employed, both generally and topically. After the hardness and compass of arterial action have been sufficiently reduced by general bleeding, if inflammatory pain and tension should remain in any part of the first passages, more blood should be freely and perseveringly taken, either by cupping or leeching, from the abdominal surface, as near to the part affected as may be convenient. Topical bleeding at the advanced and unyielding periods of visceral inflammation, promises to be more effectual in overcoming the disease, than topical stimulus in the way of counter-irritation. Indeed, it would appear, from considerable experience, though perhaps not sufficient fully to warrant its invariable adoption, that cold, topically applied over the region of the visceral cavities, in inflammatory affections of their contents, would prove more directly and lastingly remedial than either vesication, pustulation, rubefacience, or any other mode of inducing increased vascular action on the cutaneous surface. In these cases, a saturated solution of either tartrite of potass or sulphate of magnesia, in oat-gruel or barley-water, and taken to the extent of freely evacuating the bowels, will be found the best purgative remedy, as it will procure all the discharge that may be wanting, with the least possible stimulus; which, in stomachic and intestinal inflammation, is the desideratum. If it should not be speedily operative, a similar solution may be injected clysterwise; and the patient's support during the active and perilous stage of the malady, should be exclusively confined to toasted bread and water, barley-water, and oat-gruel.

An objection, it may be presumed, may be here fairly taken against the usual preposterous mode of endeavouring to move the bowels by a large variety of purgative medicines; the united stimulus of which is surely contra-indicated by the existing inflammation, and cannot be employed without occasioning a serious aggravation of disease. Aloes, jalap, scammony, calomel, &c. may be justly regarded as too stimulating on these occasions; and should in every instance be superseded by a solution of neutral salts, rendered bland by a suitable addition of castor-oil or manna. Drastic purgatives are noxious stimulants in inflammatory affections of the alimentary canal; and are, generally speaking, perhaps more likely to induce spasmodic and insuperable constriction, than a soluble state of the first passages.

A disordered action of the stomach and bowels may be truly said to lie at the root of the greatest portion of chronic diseases incident to human nature. The digestive, chylifacient, nutrient, and transmitting, functions of the alimentary canal, are of the most vital importance, and cannot be impaired without incurring much ailment, and not suspended without an inevitable destruction of life. As transmitting organs, the office of the intestines is indispensably necessary to both health and life. Their digestive and nutrient functions are equally requisite for the support and continuance of existence. The investigation that should clearly ascertain the share which a disordered state of the digestive organs had in producing most of the chronic, and many of the acute, affections arising in the animal economy, would be of inestimable practical worth ; as it would directly point to the chief cause of occurring diseases, instead of allowing of conjectures concerning them, that often prove to be seriously erroneous. Misapprehensions of the nature of disease are deplorable practical evils, as they at once preclude the possibility of affording relief, and may greatly exasperate the existing difficulty. Diseases of the first passages, whether founded in inflammatory action, in deficient tone, in disturbed habit, in vitiated irritability, in distempered sensibility, faulty secretions, or in either excessive or insufficient evacuations, are objects requiring the nicest discrimination, and the most precise adaptation of appropriate treatment. To say that a morbid state of these organs, however circumstanced, requires a free use of purgative medicines, would be to authorize the abuse into which this mode of remedy not infrequently falls.

Due attention to the history of diseases in which the stomach and bowels sooner or later become prominently affected, will show that, in by far the greater number of instances, the origin, the progress, and eventual establishment, of the disease, were principally, if not solely, referable to a disordered state of these organs. Febrile affections, whether from general or particular causes, very commonly either derive their origin, or owe their long continuance and obstinacy, to an unnatural condition of the first passages. It is enough that these important parts should imperfectly execute the several offices with which they are respectively connected, not only to induce positive disease in their own immediate sphere of action, but to awaken morbid sympathies in any or all the other vital organs. Palpitation at the heart, oppressed respiration, head-ach, pains in the ligamentous and tendinous structure, and also in the muscular fibre, disposing to spasmodic irregularity of action, are among the direct sympathetic effects of a morbid state of the stomach and bowels. Indeed, every action of both life and health is so intimately allied to an undisturbed condition of the first passages, that most of the internal, as well as external, ailments that present, may be justly said to be more or less influenced by their existing state. The most untractable diseases in surgery, whether tumors, ulcers, or even osseous enlargements and incurvations, have often the cause of their unyielding inveteracy lurking in a morbid derangement of the digestive organs ; and can only be remedied by correcting the faulty state of these parts.

The salivary, the gastric, the biliary, the enteric, and pancreatic, secretions, are indispensable agents in the healthy process of digestion. These are very apt to be vitiated when the secerning actions by which they are generated, are, by any course, rendered unnatural. Any considerable deviation from the natural state of either of these fluids, would unfit it for the decomposing and re-arranging uses to which they are severally destined in the animal chemistry of nature. If the gastric juice is not the precise product intended, it is an offensive fluid: the same may be said of the bile, and other ingredients in the process of digestion. The disordered action producing these degenerated fluids, likewise awakens visceral and other sympathies over the frame, so as greatly to disorder the general health, and to preclude the possibility of its being restored until the natural actions and secretions of the disordered parts be reinstated. This very generally is to be effected by purgative medicine, conducted so as to reach the salutary object, and not to go beyond it.

The chief indication to be regarded in the use of purgative medicines, is to excite the peristaltic power of the intestines sufficiently at once to secure a due transmitting action on the contents of the intestinal canal, and a healthful course of alvine secretions. When these salutary objects have been obtained,—when the digestive, the assimilative, nutrient, and evacuant, functions of the first passages have been established, there can be no further occasion for subjecting these parts to the influence of cathartic medicine. The morbid condition and effects of constipation, torpor, and noxious sympathies, must be overcome, before a restoration of the healthy state either of the digestive organs or of the system at large, can be produced. Continued excitement, for the several purposes of inducing additional determination of the circulating fluids, increased action, and improved energy, on the alvine viscera, is often required in affections of the first passages. These various effects do not admit of being at once accomplished. The endeavour for realizing them must be therefore pursued with a perseverance commensurate with the difficulty and importance of the object to be obtained.

The necessary distinction to be kept in view, in the use of purgative medicine in diseases more particularly of the digestive organs, is, that the prevailing debility, either of the system at large or of the first passages, is more effectually overcome by cathartic excitement than by tonic influence. The stimulus of purgative action at once energizes the languid and torpid state of vital power on these occasions, and induces actions and discharges that directly conduce to invigorate and amend the weakened and diseased state of the parts subjected to such excitement. The digestive organs have for the most part secreting functions to perform; and, if these are not aided and promoted in the various diseases to which they are liable, no radical amelioration of the morbid state is likely to be produced. Any medicinal influence that may have the effect of simply stimulating, in the calculation that the low ebb of vital power may be thereby raised and improved, would be more likely to increase and confirm the existing debility, by exhausting the feeble remains of vital power, and rendering a restoration of the healthful state additionally difficult. The salutary action of purgative medicine, properly restrained and regulated, does not therefore consist in weakening, but, on the contrary, in strengthening, by re-producing that natural state of alvine function on which health and strength essentially depend.

The correct discrimination on which the use of purgative influence should be substituted, in cases of alvine weaknesses and disordered secretions, for the direct adoption of tonic treatment, is of the utmost practical worth, because it has a tendency to remove disease, instead of locking-up and establishing it. But this great benefit is apt to be converted into a proportionate evil, by being heedlessly carried beyond the bounds by which its useful operation is limited. In the use of purgative medicine, it is warrantable to extend its influence to a full and adequate correction of all that may be amiss in the evacuant, digestive, assimilative, and secreting, offices of the intestinal canal. When these amendments have been effected, it will be time to desist from a further prosecution of the remedy; lest, by over-acting, the intended purpose of renovating lost power and function should be defeated by the morbid sensibilities and irritabilities, and even positive abrasion, of the mucous surface, that are apt to result from an excessive employ of cathartic medicine.

Torpor and insufficient action, from various causes, are apt to prevail in the first passages: on these occasions, the secreting, the digestive, and transmitting, offices of these parts, are either excessive, deficient, or vitiated. A long continuance of this state will superinduce the inveteracy of habit, than which a more untractable influence cannot obtain in the animal economy. To subdue this morbid condition of the stomach and bowels by a due use of evacuating stimulants, is indispensably necessary, before the natural energy and regularity can be restored. When this is effected, no further warranty can remain for longer employing this description of medicine. Were it to be extended beyond the salutary point of relief, it would tend to excite unhealthy action, to exhaust vital power, and finally even to disorganize the structure of the surfaces on which such protracted influence may be exerted. The advantages resulting from a judiciously-directed use of purgative medicines, are undeniably great; but these benefits are not derivable from their inordinate and indefinite employ. The determination or additional afflux of circulating fluids to the intestines, produced by cathartic medicine, removes torpor, and awakens the degree of vital exertion that is necessary to a healthy performance of the complicated and important function of the digestive organs. The system at large sympathizes with the state of the stomach and bowels: if the latter be disordered, the former will partake in the ailment. Thus, in every variety of malady, a duly active state of the alimentary canal is essential to the recovery of health.

In most diseases, it has long been the fashion to refer the chief cause to the stomach and bowels: these organs are evidently concerned, either originally or sympathetically, in a very large proportion of the diseases that occur; it will be therefore requisite, to endeavour to adjust the irregular state of these parts at the earliest period, and in the most efficient manner. The aspect of the discharges is the best criterion by which to be guided: quantity is also an object worthy of notice. Both the bulk and quality should be such as is consistent with the healthy state. When this has been fully established, the purgative indication is at an end; and, if it be carried farther, affections of inordinate excitement, colliquative discharges, mucous abrasion, and even derangement of organic structure, may be produced. Instances, it may be presumed, often occur of the purgative practice having been pushed beyond the salutary limit, and by which serious mischief has been incurred. Numerous cases of the kind are within my own immediate knowledge, in which digestive derangement, and a consequent train of nervous disease, founded in distempered and preternaturally increased sensibility, have resulted from an indiscriminate and excessive use of purgative medicine.

The object to be effected by cathartic medicine, is more to energize and restore tone to the weakened and torpid action of the first passages, than to induce evacuation. The evacuations that are successively procured neither result from excessive accumulations, nor are they originally ill-conditioned; but they are the product of disordered secretions and imperfect digestion, and are incessantly generating and amassing, whilst the diseased or unnatural action of these parts continues. When, therefore, this vitiated state has been corrected, nothing more can be beneficially done by evacuation. Adequate nutrition, air, exercise, temperance, and regularity in all circumstances that may be conducive to the general health, will best aid in establishing and preserving the acquired benefit.

Purgative medicines protracted beyond the point at which

4

they remove what would be offensive to retain, and at which they subdue diseased action, and tend to reinstate that which is healthy and natural, must necessarily be injurious. Numerous instances have occurred of the deleterious effects of unduly stimulating the intestinal canal by cathartic substances. The peristaltic power has been impaired, so as to induce either diarrhœal atony or unexcitable torpor; the villous coat has been either inflamed or abraded; the mucous secretion, instead of being a bland sheathing product for lubricating and protecting the interior of the intestines from the occurring acrimony of their feculent contents, has been so increased as to assume an unnatural arrangement, and to become thin and acrid, abounding with the saline qualities of the circulating juices. In these circumstances, it is hardly conceivable that more distress can be experienced, as the result of an immoderate, inapplicable, or abusive, employ of a valuable remedy. To overcome the diseases of torpor, faulty secretion, deficient transmission, and unnatural sympathies, is an extent of remedial influence fairly falling within the legitimate scope of cathartic excitement. The most important benefits have been afforded by its judiciously-regulated action, in every variety of febrile affection, in nervous diseases, in visceral maladies, in local ailment on the exterior of the frame, whether consisting of tumefaction or ulcerative solution of organic structure, and more especially in despeptic disorders, and in the endless train and diversity of their morbid sympathies. The use of the remedy will be shown in these numerous occasions, by its being accurately adapted to existing circumstances, by its being directed to the removal of what is evidently a state of disease, and rigorously limited to that salutary purpose. If it be urged further, and a warranty be assumed from the manifest benefit resulting from it for carrying it to an indefinite length, the worst effects of exhaustion, both general and local, of inflammation, abrasion, and ulceration, may be produced. A few deplorable cases of this description are in my reference, in which the medical evil was cruelly realized, of rendering the remedy worse than the disease.

Therapeutic medicine is under great obligations to Dr. Hamilton, for his intelligent exposition of the use of purgatives. Relying on the authority of correct observation, he has proposed, with fearless confidence, the employ of purgative medicine under circumstances, and to an extent, that had been previously unexampled. Facts justified him in recommending what he was sure would prove advantageous; and he has been amply borne out by the experience of others, in the high opinion he gave of the utility of purgative medicines in diseases which had before been treated very differently.

The first passages either at once originate or continue a large proportion of the diseases incident to human nature: the curative indication, therefore, should be watchfully directed to those parts of the system; and, as their functions are complicated and connected with digestive, secreting, and evacuating, actions of great variety, it is reasonable to suppose that they are peculiarly liable to disease, and especially requiring an adequate application of efficient remedies.

In all ages, from the remotest records of medical practice to the present period, the importance of purgative medicine has been recognized and acted on. The humoral pathology induced a strong persuasion of the necessity of such mode of attempting relief in most cases of disease. The peccant humours, as they were termed, were to be summarily expelled by the intestines; but, when this seemed to be contra-indicated by the impaired strength of the patient, the skin, the kidneys, and every other emanatory, were successively put in requisition, for the purpose of eliminating from the system its supposed corrupt and noxious juices. These refinements in the work of discharging offensive matters hurtfully diverted the attention from accomplishing the object in the only effectual way in which it could be done,—namely, by the bowels; and led to a vague, deceptious, and inefficient, treatment of disease. Had the practice been more concentered on the first passages, the beneficial effects of the Hamiltonean practice would have been perceived; and, whatever explanation may have been attached to the curative influence of such treatment, the real advantages would have sanctioned and established the practice. Authorized and illustrated as it is at present, it perhaps deserves to be regarded, under suitable limitation, to be the most safe, the most efficient, and the most salutary, mode of treatment that could be adapted for the diseases to which it is applicable.

*Taunton; October* 18*th*, 1819.

